# An investigation of modelling and design for software service applications

**DOI:** 10.1371/journal.pone.0176936

**Published:** 2017-05-10

**Authors:** Maria Anjum, David Budgen

**Affiliations:** 1 Lahore College for Women University, Department of Computer Science, Lahore, Pakistan; 2 Durham University, School of Engineering & Computing Sciences, Science Laboratories, South Road, Durham DH1 3LE United Kingdom; Southwest University, CHINA

## Abstract

Software services offer the opportunity to use a component-based approach for the design of applications. However, this needs a deeper understanding of how to develop service-based applications in a systematic manner, and of the set of properties that need to be included in the ‘design model’. We have used a realistic application to explore systematically how service-based designs can be created and described. We first identified the key properties of an SOA (service oriented architecture) and then undertook a single-case case study to explore its use in the development of a design for a large-scale application in energy engineering, modelling this with existing notations wherever possible. We evaluated the resulting design model using two walkthroughs with both domain and application experts. We were able to successfully develop a design model around the ten properties identified, and to describe it by adapting existing design notations. A component-based approach to designing such systems does appear to be feasible. However, it needs the assistance of a more integrated set of notations for describing the resulting design model.

## Introduction

The software service paradigm—sometimes termed *Software as a Service* [1] when taking an implementation perspective, or *Service Oriented Architecture* (SOA) [2], when being viewed from a design perspective—offers scope for the rapid development of new applications and systems by providing a model for reusing (distributed) implementation elements. Services can be viewed as a form of component-based system, using a very constrained but powerful form of interaction between the components, and with some components being provided by external agencies. However, guidelines for good practice in the *design* of such systems is not readily available.

To date, research into software services has largely focused upon implementation issues. We are increasingly well provided with tools and frameworks, as well as having a growing knowledge about how to employ such concepts as *negotiation* (to select a specific service) and *composition* (to integrate the services) [3, 4]. In contrast, the question of how to actually *design* such systems has received less attention from researchers, and most examples of service applications have so far been relatively simple in terms of their form and complexity [5].

However, for the service paradigm to achieve its full potential to deliver real applications across a range of domains, we need to find ways of formulating and recording design decisions about such systems in a more systematic manner. In particular, it is necessary to determine how the relevant properties of such a system may best be visualised for the designer.

This paper therefore addresses the following two research questions.

“RQ1: What properties of software services need to be represented and modelled for the design of software service applications?”

“RQ2: How well can existing software design notations be used to describe the properties identified in answering RQ1?

To address these questions, we have undertaken a phased set of studies as follows.

1. An analysis of the key properties of a system constructed using an SOA (Service Oriented Architecture), by conducting a systematic mapping study [6, 7] of definitions used for an SOA. The outcomes of this are described in [8]. and have enabled us to establish key characteristics that the design process for a service-based application needs to address.
2. A case study to examine how a ‘realistic-sized’ service-based application (i.e. not a ‘toy’ problem) can be designed around the characteristics identified in our mapping study, what forms of representation are needed to model this, and what decisions are involved.
3. A systematic evaluation of the design and related description forms used in the case study, by using a series of structured walkthroughs that involved experts from both the application domain and the software domain.

We have been able to make a good assessment of the key characteristics of SOA that are likely to need to be modelled as a part of the design process. We have also been able to demonstrate that these characteristics can be modelled using established ‘box and line’ notations. However, this modelling did require some element of reinterpretation of the notational forms, and there are good arguments for seeking to create a more balanced and unified set of modelling notations for the purpose of developing SOA applications. As such, our study can be regarded as falling into the category of *evaluation research* [9], as it involves investigating a problem ‘in practice’ (which in this case, is the design of an SOA-based application).

The paper is structured as follows. We first examine a set of background issues related to software design in general, the characteristics of service architectures, and existing research into the design of service-based applications. We describe our research methodology and its organisation and then introduce our case study, which is related to an application in energy engineering. We describe the way that the final design model was formulated and described, and report on the way that this was evaluated. Finally we assess the effectiveness of our approach and suggest some key topics needing further investigation.

## Designing service-based systems

The characteristics of software present significant challenges for the designer [10]. Added to this, as software is usually being employed to address a need in some other domain, the designer needs to combine ‘knowledge schema’ from both the computing and the application domain [11]. As with design activities in general, software design is essentially an ‘opportunistic’ process, and one that involves formulating possible design models, testing them against the known constraints, and trading off between different qualities in order to identify an acceptable solution.

For software, the ability to explore design options is also complicated by the difficulty of describing a design, since the invisibility of software means that design process has to use abstractions (usually ‘box and line’ notations) to model abstractions (the software itself). Lacking readily-visualisable forms and concepts, software engineering has adopted a rather mixed bag of ‘box and line’ notations to help record designers’ ideas, and to assist with explaining them to others [12].

### Designing service-based systems

One of the attractions of the service model is its relatively simplicity when considered from the software design perspective. The possible forms of interaction are relatively constrained, as services are self contained, and the control hierarchy is essentially similar to the traditional ‘call and return’. As such it returns the design context back to the slightly more tractable environment of earlier distributed process-based and component-based forms such as MASCOT, which was based upon a network of processes [13]. Nonetheless, like MASCOT, it still requires considerable design skill to formulate models for some of the detailed design issues such as negotiation.

As with any new implementation paradigm, anyone seeking to design a service-based application currently lacks much in the way of guidance. As designers gain experience, they might reasonably be expected to develop and codify relevant knowledge schemas [11]. Hence it may well be that in the future, the sort of mechanisms currently used to transfer schematic knowledge in software design, such as plan-driven design procedures and design patterns, will also prove to be practical for service-based models. However, until there is more extensively documented experience of developing large-scale service designs, the designer who needs to create a service-based solution is likely to lack both reusable *schematic knowledge* about how to manipulate and deploy the available resources, and also lack any established notational forms that can readily capture the essential qualities of service models.

### Software service properties

An early articulation of the software service model, also known as Software-as-a-Service (SaaS), consists of a demand-led paradigm, whereby a requirement is fulfilled by the assembly of various services, as and when needed. The distinguishing characteristic of this concept is that it separates the *possession* and *ownership* of software from its *use* [10, 14]. The SaaS model has been described as a “software delivery model” by Laplante et al. [2], where services are delivered on demand over the internet, and underpins the concept of the ‘cloud’.

To design an SOA based application, designers need the means of describing their ideas using an architectural model that provides a relatively established set of common concepts, together with notations that embody a shared understanding of how the semantics will be interpreted for the eventual implementation. To determine the key set of properties of SOA that needed to be included in a service design model, we first conducted a mapping study of SOA definitions to determine how the concept of SOA is defined and used [8]. The outcome of the mapping study was an SOA model that integrates the key elements of an SOA as shown in Table 1.

**Table 1:** Software service properties.

Identifier and Description of Characteristic
***Architecture*** Describes the overall *organization* of a system built from services as the elements, interacting through the use of mechanisms such as SOAP. **Related terms:** application architecture, architectural paradigm, architectural style, software architecture
***Binding*** The *time* at which a particular service (and provider) is chosen. In an SOA, this can be at the time of use through dynamic binding.**Related terms:** agility, dynamic binding, flexibility, loose coupling, on demand
***Capability*** The *purpose* of an SOA as viewed from an end-user perspective. **Related terms:** business functions, resource management
***Composition*** The process by which a given set of services are assembled in order to provide a single overall service that meets an end-user need. **Related terms:** choreography, integration, orchestration, service composition
***Contracts*** The mechanisms for *agreeing* upon the terms and conditions under which a service will be delivered. **Related terms:** service contracts, service negotiation
***Delivery*** The process that follows composition, whereby service *functionality* is supplied by the service providers to meet end-user needs. **Related terms:** service interaction, service invocation, service provider, service consumer
***Distributed Sources*** An SOA is implicitly capable of being created using services that are *delivered across a network* and hence that are not necessarily owned or controlled by the end-user or their agents. **Related terms:** different ownership, distributed system architecture, network environment, network
***Identity*** The characteristics that *describe* a particular service and the means by which these may be accessed. **Related terms:** broker, service discovery, service publication, service registry, service requester, service description
***Interoperability*** The mechanisms that make it possible to *deploy* services without any knowledge of their location or the means by which they are supplied. **Related terms:** connection technology, framework, hardware independent, interfaces, language independent, platform independence, standards, communication, messaging protocols
***Packaging*** The characteristics of service implementation that enable it to be treated as a unique and distinct identity. **Related terms:** component model, encapsulation, granularity, reuse, reusability, self-containment, web services
**Unclassified** **Related terms:** measurable predictions, service bus

From the mapping study we determined that:

- The definitions in the literature differ in their level of abstraction and also in their assumed context (perspective). From the perspective of a consumer, the service interface characteristics are the main aspect of interest, whereas for service providers, service implementation is an important issue. For service developers, service composition and service discovery form challenging tasks, for which they need a solution that is independent of any technological dependencies.
- The community has produced a number of different definitions of what constitutes an SOA, but predominantly, has used those from W3C, OASIS and IBM (at least, in those papers that actually referenced definitions).
- Our analysis includes only those papers that explicitly stated a source for the SOA definition used. The great bulk of publications that discussed SOA made no explicit reference to any definition of SOA,.
- There appeared to be little or no recognition of, or discussion of, the need to clarify the meaning of SOA, at least in the published literature.

### Designing applications

The end-user (or consumer) of service based applications includes both those who use an application and those who develop applications. From the service developer perspective, we can identify a number of different design approaches currently used by researchers in the SOA community. These can be broadly classified as follows.

- Those that are centered upon *service life cycles*, usually termed service oriented software engineering (SOSE). These include Offermann & Bub [15], Gu & Lago [16], Papazoglou & Heuvel [17], Erradii et al. [18], and Karhunen et al. [19]. These have covered Service Oriented software development life cycle at different levels of detail.
- Those that make use of *UML profiles*. Some of these have used UML profiles combined with modelling techniques such as model driven architecture (MDA). The studies described in Ali et al. [20], López-Sanz et al. [21], Zhang et al. [22], Warda et al. [23], Amir & Zeid [24], and Stojanovic et al. [25] come into this category. In these, the diagrammatical forms used are limited to class and component diagrams. In addition, there is extensive use of stereotypes to explain SOA features.

The relative immaturity of SOA ideas also means that it has not yet been supported by any widely used design support tools.

## Research methodology

Here, we explain our rationale for adopting a case study model as our core research method, and describe the form this took.

### Rationale for using a case study model

A positivist framework for case studies has been developed by Yin [26], who describes a case study as being an “empirical enquiry”that: “investigates a contemporary phenomenon within its real-life context” (a laboratory experiment removes it from its context); and “does so especially when the boundaries between the phenomenon and the context are not clearly evident and so may not be easily distinguished”.

So, a case study can be used to cope with the technically distinctive situation where there will be many more variables of interest than data points, which is typically the situation for studies of software design. A case study analysis therefore relies upon multiple sources of evidence, using triangulation between these to give confidence in the validity of the outcomes.

From the experience of conducting the mapping study on SOA we observed that, while the examples commonly used in the SOA literature may be adequate for illustrating the proposed methods, they are artificially constructed, lack originality and are narrow in scope, as has been observed by other researchers too [5, 27].

Espinha et al. conducted a literature survey on the case studies used in SOA research and reported fourteen case studies published in the CSMR, ICSE and ESEC/FSE conferences and also from an European S-Cube project on service based applications (SBA) [5]. They identified case studies that are too small to be representative of real service based systems such as [28] and [29], and also some that include more services, such as [30], although the details of this are not available. The same situation applies to industry-based reports. Researchers may mention that their approach has been applied to real applications, but provide no details.

### Design of the case study

We adopted an *exploratory* role for this case study, with the aim of helping future model building by identifying what the key decisions were, and how the system was modelled in order to help with these [26]. This was organised as follows.

**Fig 1:**
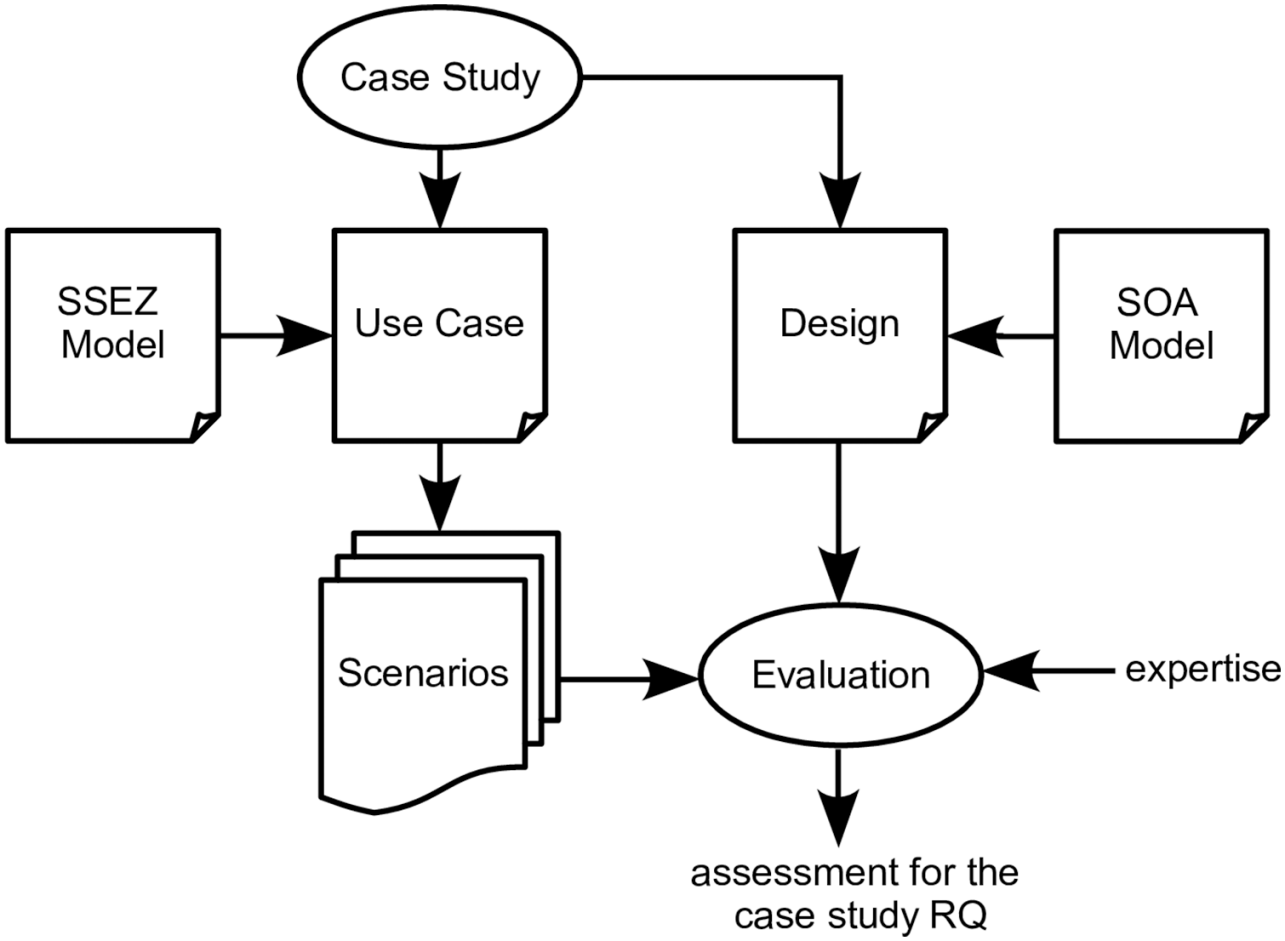
Overview of the case study design.

**Case Study Domain:** The case study was taken from the energy engineering domain as we had ready access to the resources within the school of Engineering & Computing Sciences at Durham University. Within this domain, the concept of *smart grids*, which is similar to that of a *small scale energy zone* (SSEZ), is considered an important idea in future power systems. The concept involves organisation of distributed power generation in a form that is local, and also independent of the grid. This helps with supplying power when there are major and widespread blackouts in case of extreme weather conditions such as ‘Hurrican Sandy’ in 2012 [31]. Further to this, when prediction models are integrated with power systems, this can help to remove loads from the grid by estimating future power needs in the area [32].
**Case Study Type:** While a multiple-case study could provide stronger evidence, the need for a suitably large example meant that we were limited to using a single-case study, using a ‘typical’ case.
**Unit of Analysis:** The ‘case’ (or unit of analysis) was the design for a small scale energy zone (SSEZ) control system. This is a real time system that is run by an Energy Services Company (ESCO) to manage the electrical network and fulfil energy needs in an SSEZ.
  We constructed an operational model of the SSEZ control system in the form of a use case. (To reduce scope for confusion of terms, we will refer to this as the “SSEZ use case” in the rest of this paper.) This contains electrical network information, operational goals, key factors related to control system and the data involved. This was then instantiated as a set of *scenarios*, using specific parametrisation options. The purpose of the model was to provide planning at 30-minute intervals, allowing the ESCO to change the status of its electricity generation resources as necessary, or to purchase additional resources from the grid.
**Characteristics of the SSEZ Use Case:** These were: the need for *negotiation* with providers and consumers of electricity involving use of *multiple distributed sources* of information to make decisions; and for *adaptability* of the model. The SSEZ requires information from different sources (network and service providers). The involvement of service providers requires negotiation to be present in the case study. As this is a real time system, adaptability is also an important attribute of this case.
**Data Collection:** The case study data was collected both through the use of method triangulation [33], as shown in Fig 1 and also by employing triangulation of multiple data sources. Table 2 provides details about this. These techniques were used to increase the validity and consistency of the data. In a case study, the confounding factors that may bias the result are not entirely known or cannot be controlled. This is because in a case study the researcher does not have the same control as in an experiment. Yin suggests two ways to handle this problem:
  - by conducting multiple case studies or use of multiple cases in a single case study (an option that was not really available to us);
  - by using triangulation to gather evidence in a single study (as adopted here).
**Time Period:** The time needed to perform the case study was longer than the period specified in the research protocol. One reason for this was the iterative approach of collecting data and then analysing it to identify gaps in the information. A second reason was the time required to obtain relevant domain knowledge. Because of its interdisciplinary nature, vocabulary played an important role in data collection and representation, and so clear definitions of terms were needed to ensure that any documents produced could be understood by both disciplines.
**Analysis:** The scale and complexity of the problem meant that it was impractical for us to construct an actual system, not least because this form of energy engineering is also an emerging rather than an established form. We considered two options for evaluating our design:
  - using a *simulation* of the SSEZ;
  - using *design reviews* or *walkthroughs* with expert participants.
  Since we wished to gain insight into the design process overall, as well as the effectiveness of the design notations employed, we adopted the second approach, since a walkthrough can provide qualitative information and insight.

**Table 2:** Data collection.

Method Triangulation & Data Triangulation
***SSEZ Use Case:*** The data about the use case was collected through interviews with domain experts and the study of supporting documents that include research papers, technical reports, and thesis. The information collected in one interview session was used in the next with some additional documents to collect feedback. This was important to identify inconsistency in the collected data and to make sure that the domain information has been understood correctly. The interview sessions (formal and informal) with domain experts helped with understanding the domain and the supporting documents provided sources for its vocabulary. This was mainly a process of requirements elicitation.
***Design:*** The SOA design model constructed as the part of our case study provides details about the service and functional components created from requirements. The design elements are presented through abstract diagrammatical forms.
***Evaluation:*** The walkthroughs and interviews were conducted as part of case study for the validity of use case and the design. The data for evaluation was collected both from walkthrough sessions and also from interviews with participants. The data about walkthrough sessions contains details about the review. The data was recorded in audio and video files. The feedback about walkthrough process, and design presentation was collected through interviews.

### The energy engineering use case

The SSEZ use case describes the situation where an Energy Services Company (ESCO) is maintaining its electrical network by generating electricity through renewable energy resources and trying to avoid the use of conventional power where possible. The main objective for the ESCO is to provide electricity to its customers in an efficient, reliable and cost effective way. To achieve this target, the ESCO needs to be able to predict demand and generation for its electrical network; to take decisions, where required, about the buying and selling of energy, as well as when to adopt an islanding mode. Islanding refers to the situation where distributed generator(s)(DG) continue to maintain the network voltage and frequency within regulatory limits to a location even after disconnection from the power utility [34]. and to take decisions about demand side management (DSM). For this, the ESCO has to gather and process network and commercial data from different sources and use this to make real-time decisions.

**Fig 2:**
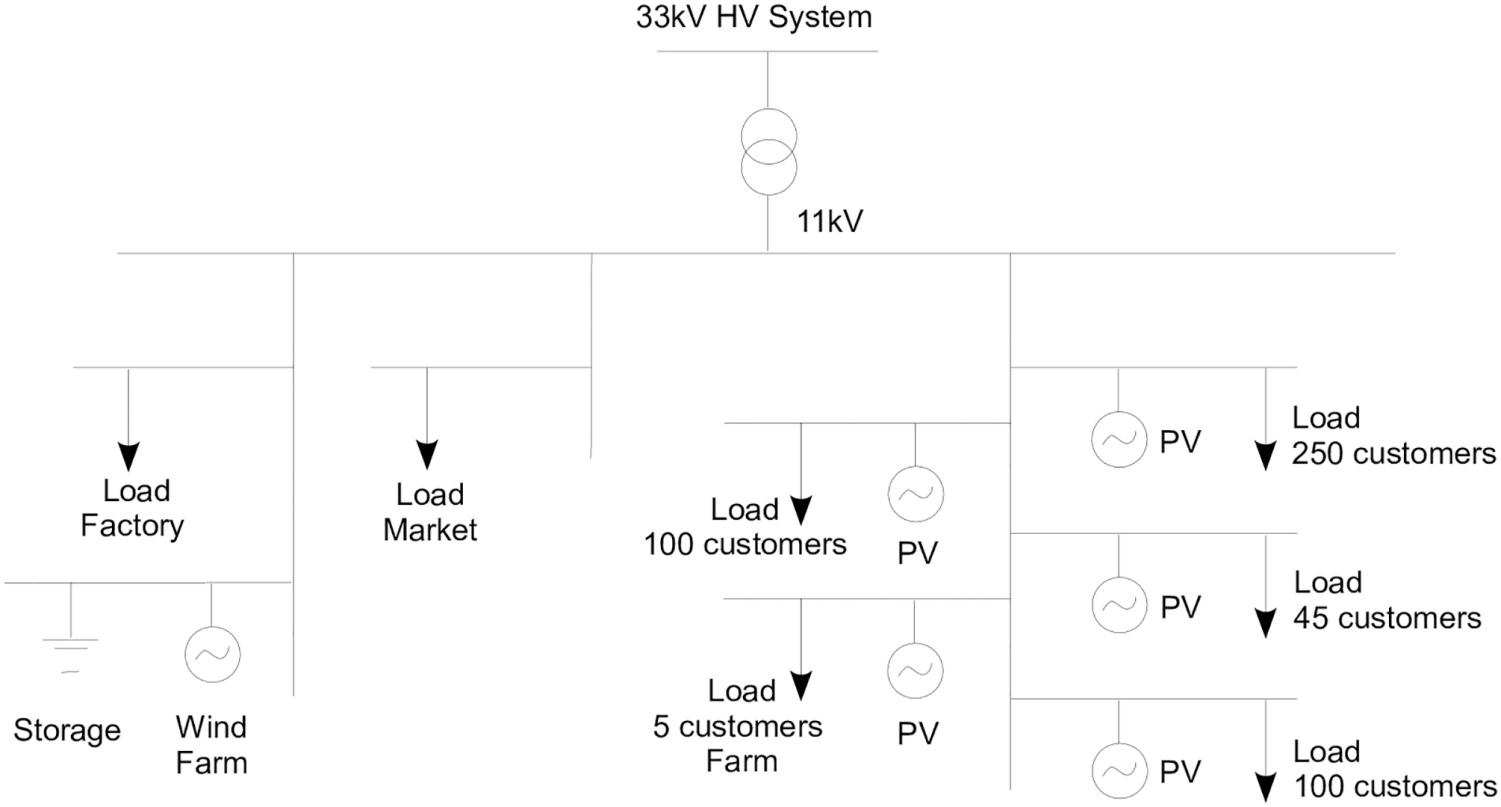
The electricity network used for the SSEZ use case.

The SSEZ use case defines an operational model for the SSEZ. An SSEZ is defined as a controllable low voltage distribution network (LVDN) that consists of a number of different small scale embedded generators (SSEGs), distributed energy storage units (ESUs) and customer demands [35]. Fig 2 shows the basic configuration that we used for the SSEZ use case (for practical purposes, we needed to employ a specific configuration in order to be able to resolve some of the design choices). This includes a number of generating sources (wind farm, photovoltaic panels), a storahe device, and some specific loads that would be expected to have different profiles of power use (industry, domestic, commercial). Fuller details are available in [36].

### Limitations of the case study

Case study research, especially when using single-case forms, does have some implicit limitations. As Yin [26] observes, even having two cases can provide more compelling evidence and also an element of replication.

Perhaps the biggest challenge for single-case forms is how ‘representative’ the choice of the case is. So, given that the scale involved would have made it difficult to use other than a single-case form, we need to consider whether our choice of the SSEZ use case was a suitable one for an essentially methodological study. From a service perspective, the SSEZ control does involve a good mix of ‘local’ services and services that can be provided by other suppliers. It is certainly more ‘real world’ than the examples considered in other studies, but there does remain some risk that it exhibits some features that may have unduly influenced design choices to a degree that would not occur for most other systems.

## Conducting the case study

The main actions of the case study itself involved:

1. Identifying how the characteristics of the SSEZ use case could be matched to the characteristics established for an SOA.
2. Developing a software service-based design, assuming a set of likely services as well as those already existing.
3. Conducting an evaluation of the design using both domain experts and software engineering experts.

### Matching the characteristics

To confirm that the use of an SOA was appropriate for the SSEZ use case, we began by interpreting the SSEZ use case in terms of the characteristics described in Table 1, but omitting *delivery* and *packaging* as not being relevant in a design context.

- **Architecture:** Control of the SSEZ requires information to be collected by a centralised element. This means its structure can take the form of a ‘tree’ where lower level services (network information) provide information for higher level services such as a control service.
- **Binding:** The state of the SSEZ needs to be reviewed at regular intervals, to take account of current values and forecasts for demand, provision and weather. Each review may involve a different set of information sources, especially as resources providing generation and storage may be added and removed from the current profile of the SSEZ. The model of late (runtime) binding provided in an SOA is therefore particularly well-matched to this need.
- **Capability:** The meaning of this is the same for both an SOA and for the system as a whole, and is concerned with the overall functionality. For an SSEZ, it is related to the ability of the software to use available information to perform the necessary resource management, by modelling demand and provision for the next period of time and then plan any changes accordingly. That in turn is related to the set of algorithmic models used for prediction, the state of the system at any time, and the forecasts for the next period.
- **Composition:** Composition involves bringing together multiple sources of information to facilitate a decision. An example of this in an SSEZ might be the use of information from a wind farm about its current output, together with forecasts of likely demand and a weather forecast, to decide whether to increase or decrease provision from other sources. Composition is a core feature of a software service model, which also provides the means to select the source of a service (e.g. weather forecast) when the request is issued (late binding), and hence this characteristic makes a service solution well matched to needs of an SSEZ.
- **Contract:** The contracts are rules of engagement with other services or between service provider and consumer. In the case of an SSEZ, contracts for different types of forecasting services may address factors such as the granularity of data and service availability. If the rules of engagement change, then any subsequent re-negotiation may result in a change of service provider.
- **Distributed Sources:** The SSEZ is well matched with this implicit feature of an SOA. The data coming from generation, demand and weather is already coming from distributed sources with different ownership.
- **Identity:** The characteristics of demand, generation, weather and their forecasts are different and are accessed through different means. Collectively, these characteristics help the generation forecast service to select the most suitable service provider for its processing.
- **Interoperability:** For an SSEZ this is an important feature as its elements need to be able to communicate information with each other in a consistent framework. To ensure interoperability, the electrical power industry is working on two standards: Common Information Model(CIM) and IEC 61850. In an SOA, interoperability is provided by a combination of XML-based messaging forms and an ontological model that provides the necessary semantics. This is clearly highly consistent with the above.

Having confirmed that an SOA was capable of modelling the key issues for the SSEZ use case, we then proceeded to develop a design for this.

### Creating a design model

A designer’s goal is to transform what is usually an incomplete and ambiguous set of requirement specifications into a high level system design, expressed in formal or semi-formal notations [37]. The process of design is a creative task, and is commonly performed by using an ‘opportunistic’ strategy, in which the design decisions are driven by the ‘unfolding’ understanding of the requirements as well as the emerging design model [38].

The design produced at the end of such a process will be the outcome from multiple iterations of of the activities involved in exploring and exercising the design model, as it is usually not possible to identify all of the features in the first round. Also, the identification of one aspect may lead to the discovery of the need for a new service or change some features of one already identified. Therefore, there is no specific order for performing the type of design activities discussed below and they are usually interleaved to some degree.

#### The design process

While describing the design for the SSEZ use case, we have also used the term ‘*service-based control system (SBCS)*’; which means ‘*the software system that is going to control the activities of the SSEZ*’.

Beginning from a set of brainstorming sessions, and from a process of iterative design, we were able to identify the following design attributes for the SSEZ problem.

1. **Identification of Functional Components** (functional decomposition of the system)—We partitioned the system into a set of functions through which the overall system functionality can be realised. This was based on the well established concept of ‘separation of concerns’ [39].
  The goal of this activity was to identify the main functional components and the outcome was a list of operations through which the overall system functionality can be realised. Nine main functional components were identified, as shown in Table 3.
2. **Identification of Potential Services**—Identification of services is associated with determining how the functional components identified in (a) will be delivered. A service may provide one or more functions, and may contain a logical grouping of functions within itself. The inputs and outputs determine the extent to which services depend upon each other. This helps with planning composition of services and with construction of the workflows. Therefore the main goals of this activity are:
  - Identification of services (the functionality that is reusable and will need to be obtained from a third party or be provided as service to others)
  - Identification of service roles (what functionality the service will provide)
  - Identification of inputs and outputs of these services (which will lead to identifying of dependencies and developing a composition flow)
  For the SBCS, the goals achieved through this activity helped to develop the possible interfaces for the services (which can be realised through a class diagram). They also helped to define the parameters needed for service invocation.
  The output from this activity was a list of identified potential services, including their roles with possible inputs and outputs, as shown in Table 4.
  Note that the controller that coordinates the process was not considered to be a service because it does not offer a service outside of its own system. Instead, it makes use of the services listed in Table 4. Therefore, F2 and F6 are part of the controller and are not represented as a service. Also, the service (*S*_*HD*_) responsible for providing information about historical demand is identified as part of the demand prediction service.
3. **Functional Traceability**—The functional components were allocated to the services by means of a ‘traceability table’. This specified the mapping between functional components and the candidate potential services. The purpose of using functional traceability was to document how a function was provided by specific services. This modelling step helps both to identify service granularity and to determine how cohesive they are. To identify which service was contributing to the realisation of a particular functional component we also used a functional ‘realisation’ table. The purpose of these two tables was to:
  1. track which service was being used to provide specific functionality and where this functionality would be available locally.
  2. identify the services that provide inputs needed to realise the functional component, where this is not part of their operations.
  The result of this activity was a mapping between identified functional components and candidate services which was again represented through the use of tables.
  The traceability table shown in Table 5, represents the role that each service plays in providing the functionality listed in Table 3 for the SCBS. A cross indicates the involvement of a service in a function and its absence means that the service plays no role in the realisation of that function. The functional components, F2 and F6 are not provided directly by services and are part of the controller, therefore no crosses are included in these two columns.
  Table 6 shows how the services have been mapped to functional components in order to represent how the functionality of these functions is realised. These functions are not part of a service, rather they use information from the services to perform their tasks.
4. **Service Interactions**—The profile of interactions between services was described by using a tabular structure where services are ordered on the horizontal and vertical axes, in the same order. The purpose of interaction could be to get data or to provide a function for other services.
  The interaction of services can also be synchronous or asynchronous. This is an important design choice and depends on the requirements of the system, since it affects the way services are implemented. The important factor is that services are considered stateless and scalability is considered an important factor in SOA design. However, the application domain has its own constraints that also effect the design choices. For the SBCS, the result of this activity was a table structure representing the service interaction pattern.
  In Table 7, services are listed along the x-axis and the y-axis in the same order, and the table shows where services interact with each other, including the controller (represented by C). The cross indicates service interactions and absence means no direct communication between services. We found this representation useful in terms of identifying how services interact and depend upon each other.

**Table 3:** Functional components (modules).

Functional Components	Roles
Get system states (F1)	Report SBCS states from three different sources (generation, demand and storage).
Assess power balance (F2)	Check balance in current demand and generation.
Get weather forecast (F3)	Get weather forecast for SBCS.
Predict demand (F4)	Predict demand based on current and historical data.
Predict generation (F5)	Predict generation based on weather forecast and current generation status.
Assess level of change (F6)	Check different options to assess the type of change required to maintain an energy balance.
Get market price (F7)	Get energy market price for SBCS.
Update system states (F8)	Take action by changing system states.
Update system log (F9)	Update data in the SBCS system database.

**Table 4:** Service role, inputs and outputs.

Services	Roles	Inputs	Outputs
Service to get generation output (*S*_*G*_)	Provides data from wind turbines.	-	generation output, wind speed
Service to get demand (*S*_*D*_)	Responsible for providing current demand data.	-	energy consumption data
Service to get historical demand (*S*_*HD*_)	Responsible for providing historical demand data.	-	energy consumption data
Service to get storage status (*S*_*S*_)	Provides current state of charge (SOC) and storage status (SS) for storage unit.	-	SOC, SS
Service to predict demand (*S*_*PD*_)	Provides demand prediction.	current demand, historical demand, weather data	demand prediction data
Service to predict generation (*S*_*PG*_)	Responsible for providing generation predictions.	current generation, weather data, location	generation prediction, wind speed
Service to get weather forecast (*S*_*W*_)	Provides weather data (current and forecast).	location	wind speed, temperature
Service to provide energy market price (*S*_*M*_)	Responsible for providing the energy market price.	-	buy and sell price
Service to maintains system log (*S*_*L*_)	Updates data in the SBCS database.	system states, weather data, market price	-

**Table 5:** Functional traceability.

	F1	F2	F3	F4	F5	F6	F7	F8	F9
*S*_*G*_	×								
*S*_*D*_	×								
*S*_*S*_	×								
*S*_*PD*_				×					
*S*_*PG*_					×				
*S*_*W*_			×						
*S*_*M*_							×		
*S*_*L*_									×

**Table 6:** Functional realisation.

	F1	F2	F3	F4	F5	F6	F7	F8	F9
*S*_*G*_		×				×			
*S*_*D*_		×				×			
*S*_*S*_		×				×			
*S*_*PD*_		×				×			
*S*_*PG*_		×				×			
*S*_*W*_				×	×				
*S*_*M*_						×			
*S*_*L*_									

**Table 7:** Service interactions.

	*S*_*G*_	*S*_*D*_	*S*_*S*_	C	*S*_*HD*_	*S*_*PD*_	*S*_*PG*_	*S*_*W*_	*S*_*M*_	*S*_*L*_
*S*_*G*_				×						
*S*_*D*_				×						
*S*_*S*_				×						
C	×	×	×			×	×	×	×	×
*S*_*HD*_						×				
*S*_*PD*_		×		×	×					
*S*_*PG*_	×			×			×	×		
*S*_*W*_				×		×	×			
*S*_*M*_				×						
*S*_*L*_				×						

#### Design decisions for the SSEZ

The design was developed to be independent of any implementation technology, and some key decisions associated with the SBCS design are discussed below.

**Controller:** The Controller was not considered to be a service. This is because it initiates the ‘control cycle’ periodically, and for that it needs state information of the SSEZ electrical network, have to consider technical constraints and priorities set by operating policy.
  Also, the decision to make the controller a service depends on the ESCO’s long term policy about its business. In present design, the ESCO is using third party services to facilitate its energy zone, and the controller is part of its internal system. Also, the ESCO is not selling services to anyone outside the zone. In future, if the ESCO decides to provide the functionality of the Controller as a service to other ESCOs or other SSEZs then it would need to be designed differently. In that case, the controller can be considered as a service and it will need network related information and other operating constraints. Therefore, the decision to consider a particular element of functionality to be a service is appropriate when there is more than one consumer of the service.
**Registry:** In our design, the service registry was assumed to be owned by the ESCO itself. This decision was made because of the nature of the application domain. We regard it a domain specific decision because in a control system, time is an important factor and using a third party registry could be a possible constraint in accessing services in time.
**Market Service:** The Market service was represented as a single service provider by considering the present situation where the U.K. national grid forms the only distributor.
  To make this scenario more complex, it can be assumed that this service is offered by different service providers, as in the case when a neighbouring ESCO offers its service. This scenario involves important decisions by the controller and use of the registry.
  First, it adds further choice for the *Controller*: such as whether to buy ‘green energy’ (produced from reusable resources) from a neighbouring ESCO, or to use ‘brown energy’ from the national grid. Second, where there is more than one possible service provider for generation, we need to add the relevant information to the registry, and the registry provides a mechanism for doing this. So two levels of decisions will be involved for buying energy. First, using the weather to select green or brown energy, second, selecting the right service provider. In doing so, the time required to complete this activity needs to be critically analysed.
  In this design, we sought to generalise our use case by employing a scenario where we have a fixed service provider for some services and then more than one provider for other services. Also, we allowed for the possibility of having different contracts with these providers, where these could be long as six months to one year. Therefore, in the case of long or short term contracts, the time constraint discussed above could be eliminated. This feature is related to the application domain and depends on the business strategy of the ESCO.
**System States Service:** This service is logical and internal, responsible for collecting and providing network status to the controller.
**Weather Service:** Weather data includes temperature, wind speed, wind direction, solar irradiance, cloud conditions, along with information about the area. The level of detail (usually with regard to time intervals) offered by each weather service may be different.
  We made the decision to include the weather service early in the process, because on each cycle, the SBCS needs to model two situations regarding the state of the SSEZ i-e. present and future. The current weather information is treated as being part of the current system state and is also used to maintain history about the condition of the zone. The weather forecast is required for predicting the future state of the zone and assessing the effects upon this state of scheduled calendar events such as sport fixtures and major holidays that could change demand patterns.
  The ESCO takes generation prediction and demand prediction services from a third party. The level of detail in these services could differ as services could be very simple ones that address weather information, demand and generation values and provide predictions for these or they could be realised as more powerful services that require network information, and location information along with current demand and generation data.
  At a later stage, the ESCO might consider providing these services as part of its business policy. This decision will effect the way prediction services are accessed. A prediction model consisting of these services will need to be developed and weather services will become part of this model.
**Network Configurations:** These are technical constraints that include low level details about the electricity network (such as assets information) that are not included in the design. This role is associated with the *Controller*.
**Operating policy:** This is required to determine how decision making will be performed at different levels. The policy determines the priorities that the *Controller* uses when making decisions. If policy is that the SBCS will try to remain self sufficient then in case of an increased demand in the zone, the first priority for the SBCS will be to seek to defer this. However, if the highest priority is to meet customer demand then the SBCS have to import power from the grid. In that case it also has to evaluate the use of green and brown energy and consider its policy about the use of brown energy.
**Non-functional Features** Design time non-functional attributes are considered, such as time and cost. These provide the main selection criteria for the services though the availability of the service at run time might also influence the choice made by *Controller*.

### Representing the design model

We used the viewpoint classification described in [12] to categorise the set of properties that describe the static and dynamic features of the SOA design model. To represent these, a mix of functional, behavioural, constructional and data modelling notations need to be employed.

We made use of representational forms commonly used with other architectural styles to represent the viewpoints, as there are no generally accepted notations used in SOA design. Making use of existing forms of notation has the benefits that there is likely to be some tool support available (at least, for producing the model, if not necessary for checking it), and that the interpretations are familiar. What we describe here is our final choice of representation forms, after some element of trial and error regarding the forms used. In our examples of the representational forms we have also tried to keep our descriptions of the SSEZ to the same level of abstraction as nearly as possible.

Table 8 explains the purpose of using a particular representation, and the viewpoint that it is intended to provide. Some points to note about our choices are as follows.

- Both Data Flow Diagrams (DFDs) and UML activity diagrams have been used to show the functional aspects of the SBCS. A DFD provides a ‘big picture’ of the SBCS and can aid with functional decomposition of the system. The activity diagram can be used to provide more detail about the processes.
- The activity diagram is particularly useful for representing how services interact with each other in order to perform a specific task or to realise a business process. During service composition, a workflow is generated that describes the sequence in which services will be assembled and executed. An activity diagram can be used to describe this.
- The UML class diagram serves two purposes. It can provide the conceptual decomposition of the system into services. It can also be used to model service interface information.
- The UML component diagram has been used to show how system components and services are interacting with each other. (The interfaces that are provided and required by them.) This also helps to present the physical and logical views of services.

**Table 8:** Purpose, representational forms and viewpoints.

Purpose	Representation	Viewpoint
Problem oriented view of system with its inputs, outputs and processing elements	Data flow Diagram	Functional
Service operations and relationships	Class Diagram	Constructional
System components and Service interfaces, relationships, and service providers	Component Diagram	Constructional
System flow with functional components internal and external to the system, sequencing and ordering of activities	Activity Diagram	Behavioural/ Functional
Interaction and order of interaction among services over time	Sequence Diagram	Behavioural
Overall system behaviour, interaction with services and decisions by the control	Flow Chart	Behavioural

We now examine these in a little more detail, illustrating their role with elements from the SSEZ use case.

#### Data flow diagram (DFD)

The form of DFD notation introduced by De Marco [40] provides a useful abstraction of functional nature of the overall system, and has been widely employed for analysis. For *Structured Analysis* [41], the DFD provides a well established way of representing processes, external entities, data flow and data stores using symbols that are visually distinctive—which also helps in managing the diagrammatical complexity [42].

**Fig 3:**
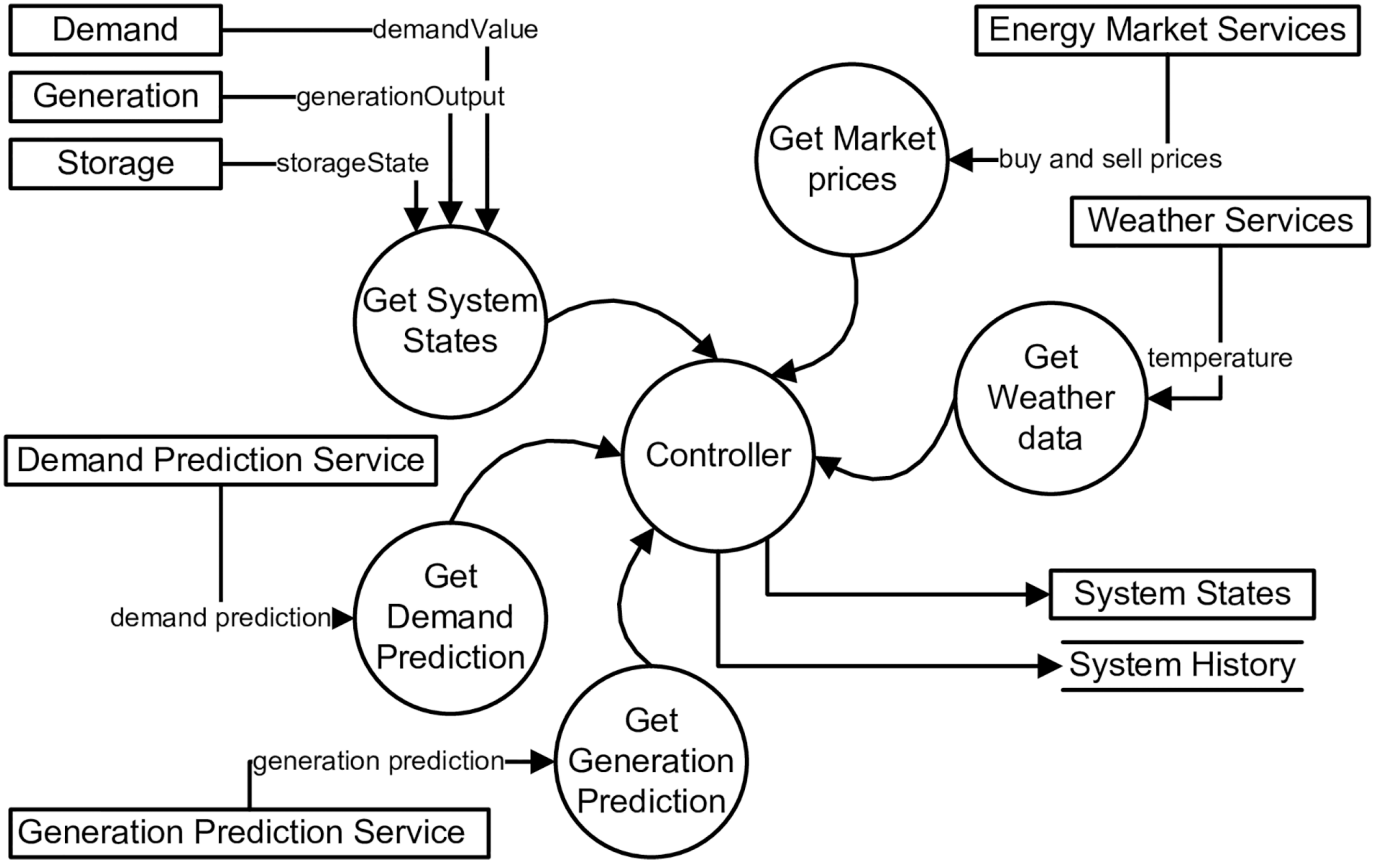
Data flow diagram (DFD) with details about entities and data.

Fig 3 uses a DFD to represent an abstract view of the SBCS by modelling the interactions between its processes, the external entities that it communicates with, and the data it stores. Information about system state is treated as three external entities that provide information about energy consumption, energy generated and energy stored in the zone. Current and predicted buy and sell prices are taken from the *Market Service*. The *Weather service* provides current and future weather data. *Demand* and the *Generation prediction services* provide the prediction values to evaluate the likely future condition of the SSEZ.

#### Class diagram

**Fig 4:**
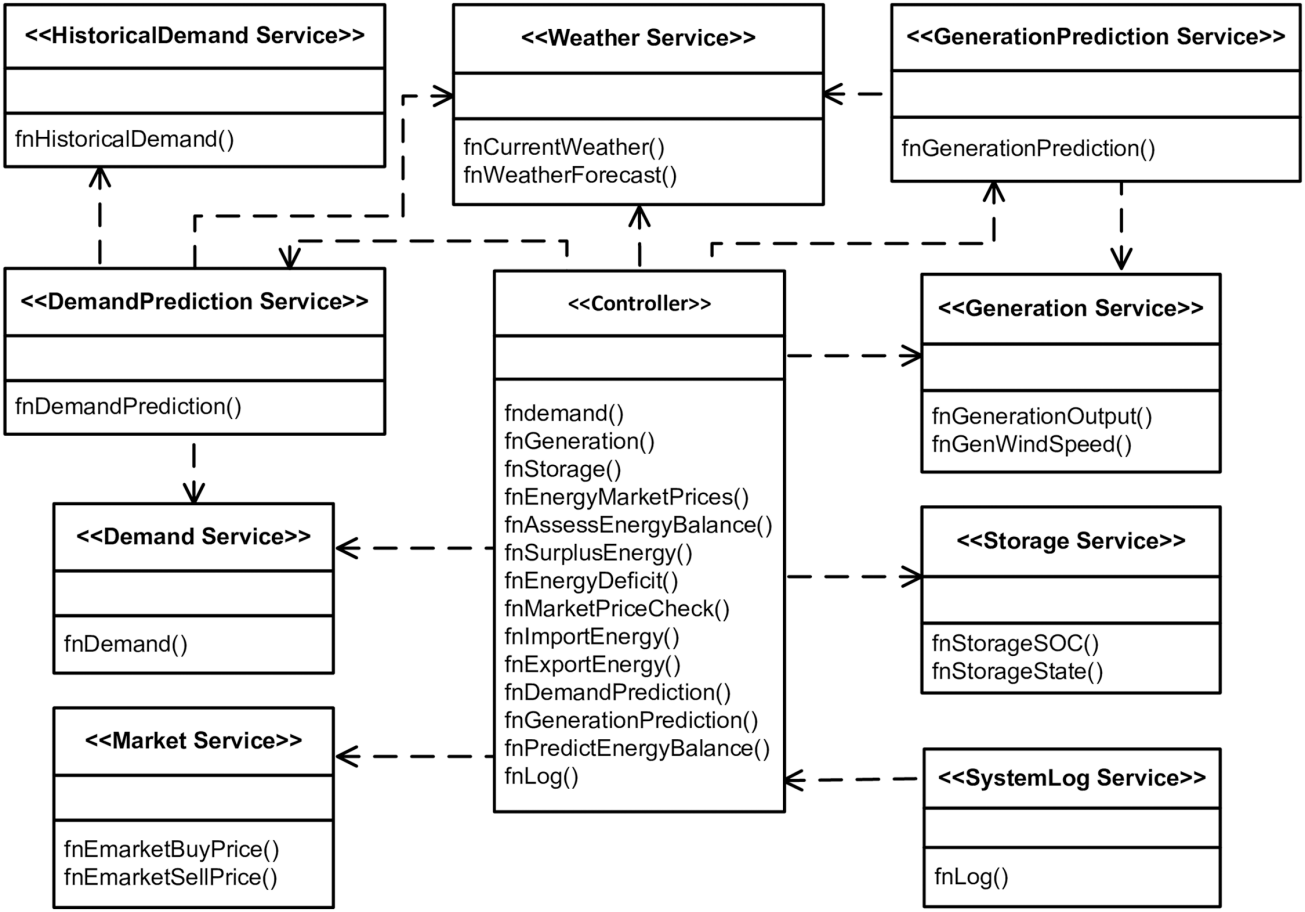
Class diagram for service dependencies and operations.

The UML class diagram was used to represent the static features of the SBCS. In Fig 4, a service model for the SBCS is represented using a class diagram. The operations that services offer and perform are listed in the rectangular boxes. The interactions among services which show their dependencies on each other are represented through dotted lines. Stereotypes have been used to give more meanings to the service model characteristics.

The *Controller’s* operations are performed internally, while other services offer interfaces designed to be invoked. For example, the *GenerationPrediction Service* takes data from the *Weather Service* and *Generation Service* and provides predictions to the *Controller*. The *DemandPrediction Service*, invokes the *Demand Service* and provides demand prediction to the *Controller*. The *SystemLog Service* is a service that is dependent on the *Controller*, which it accesses to get the data needed to maintain the system history.

#### Component diagram

The UML component diagram has been used to represent the interactions of services, their interfaces, and any dependencies between system components. This also provides information about the interfaces that are offered by services and the ones required by system components.

We have used a component diagram to represent:

- dependency among services and SBCS components;
- the interfaces provided by services and used by components;
- information about service providers, such as where a choice of more than one service provider is available. This information is helpful when we have a mix of fixed and multiple service providers. It also helps to decide whether there is a need to use the registry in the case of the availability of fixed service provider or whether it should be treated as a static binding. We consider this to be an important design decision, as this will add or remove the processing of the searching service from the registry.

**Fig 5:**
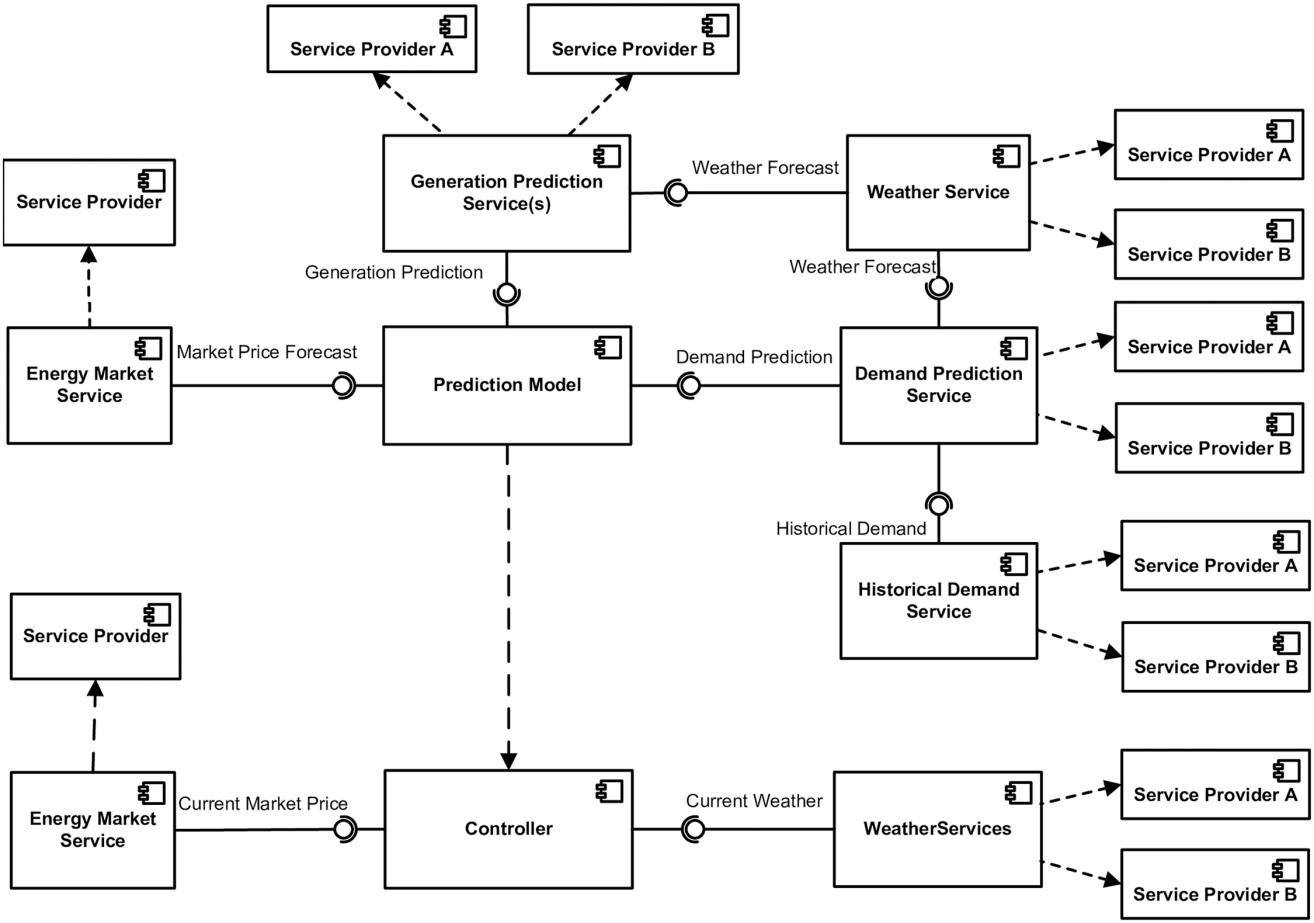
Component diagram for SBCS.

In Fig 5 we have divided the SBCS into two main components: the controller and the prediction model. These two components are internal to the SBCS and use other services.

The *Energy Market Service* has a fixed service provider. This service interacts with controller to provide current market buy and sell price.

The *Weather Service* is shown as having two possible service providers. For this reason it is necessary to add a registry to the SBCS functional components. The registry component will maintain information about the available weather service providers and any non-functional information that includes the time and cost of using these services. Further, the weather service used here is also providing current weather conditions for the controller. So each time the controller accesses this service there is the possibility that a different service provider might be selected.

The services interacting with the prediction model also have multiple choices of service provider. It is possible that the *Weather Service* that provides current weather to the *Controller* will be different to the one that is used by the *Generation Prediction Service* and the *Demand Prediction Service*. Again, this means that each time the *Controller* executes its process, a different set of services might be selected. The dotted arrow going from the prediction model to the controller represents the decision that this component is executed as part of the controller component.

#### Activity diagram

The UML activity diagram provides a workflow-oriented view of a problem by capturing both functional and behavioural aspects. We have used an activity diagram to represent the overall flow in the SBCS, and for different scenarios that show behavioural aspects.

**Fig 6:**
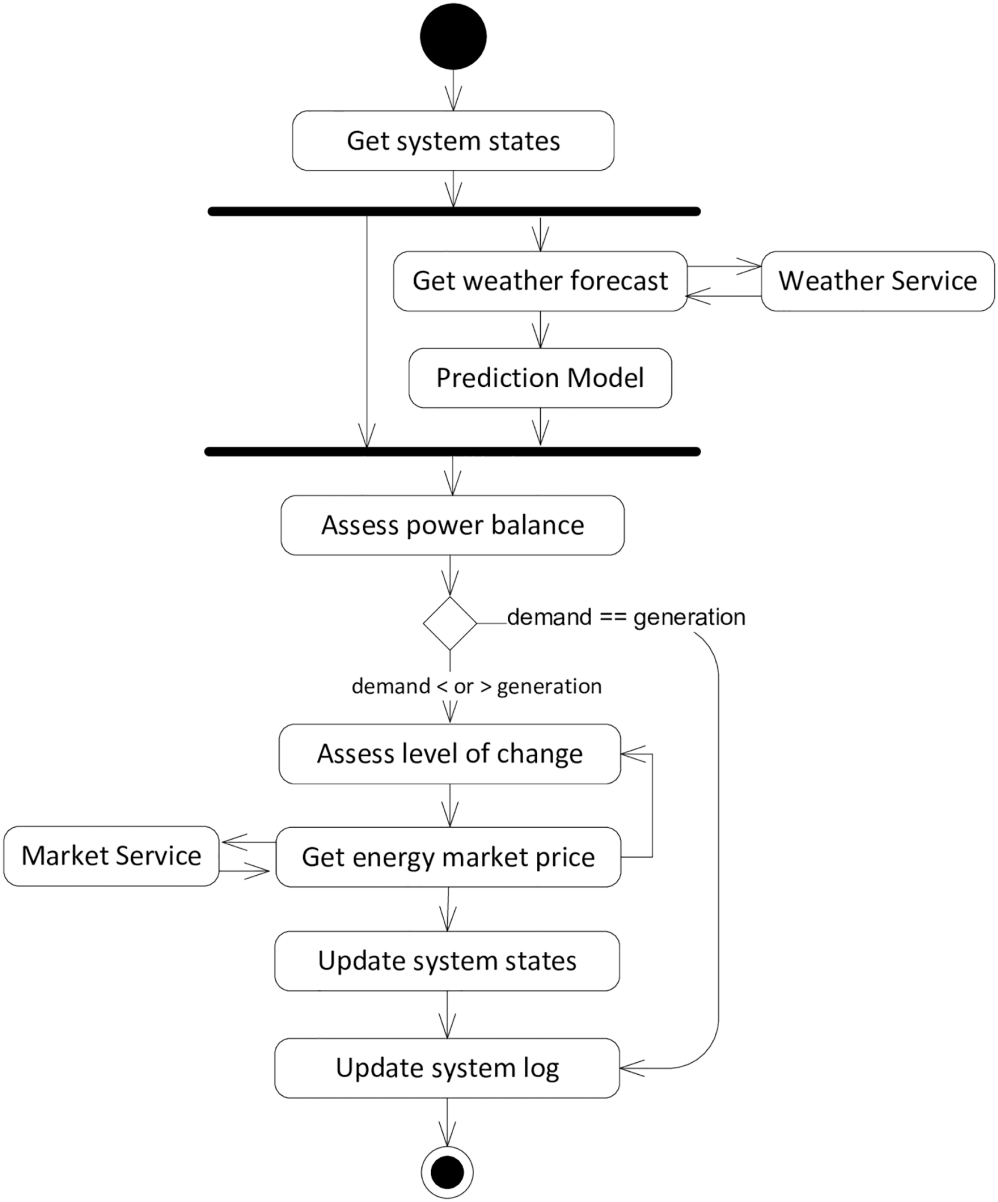
Activity diagram to show overall SBCS flow.

Fig 6 describes the main flow of the SBCS. The process starts by accessing the SBCS states (consumption, generation and storage), and the weather forecast data as well as assessing the current power balance in the SSEZ. If no changes are needed, the system simply updates the log and process ends. If there is a difference between the current state and the predicted demand and generation, the process examines the system states and market prices to determine what options are available, and makes a choice from these.

#### Sequence diagram

The role of the UML Sequence diagram is to represent the communications that occur between services over time.

**Fig 7:**
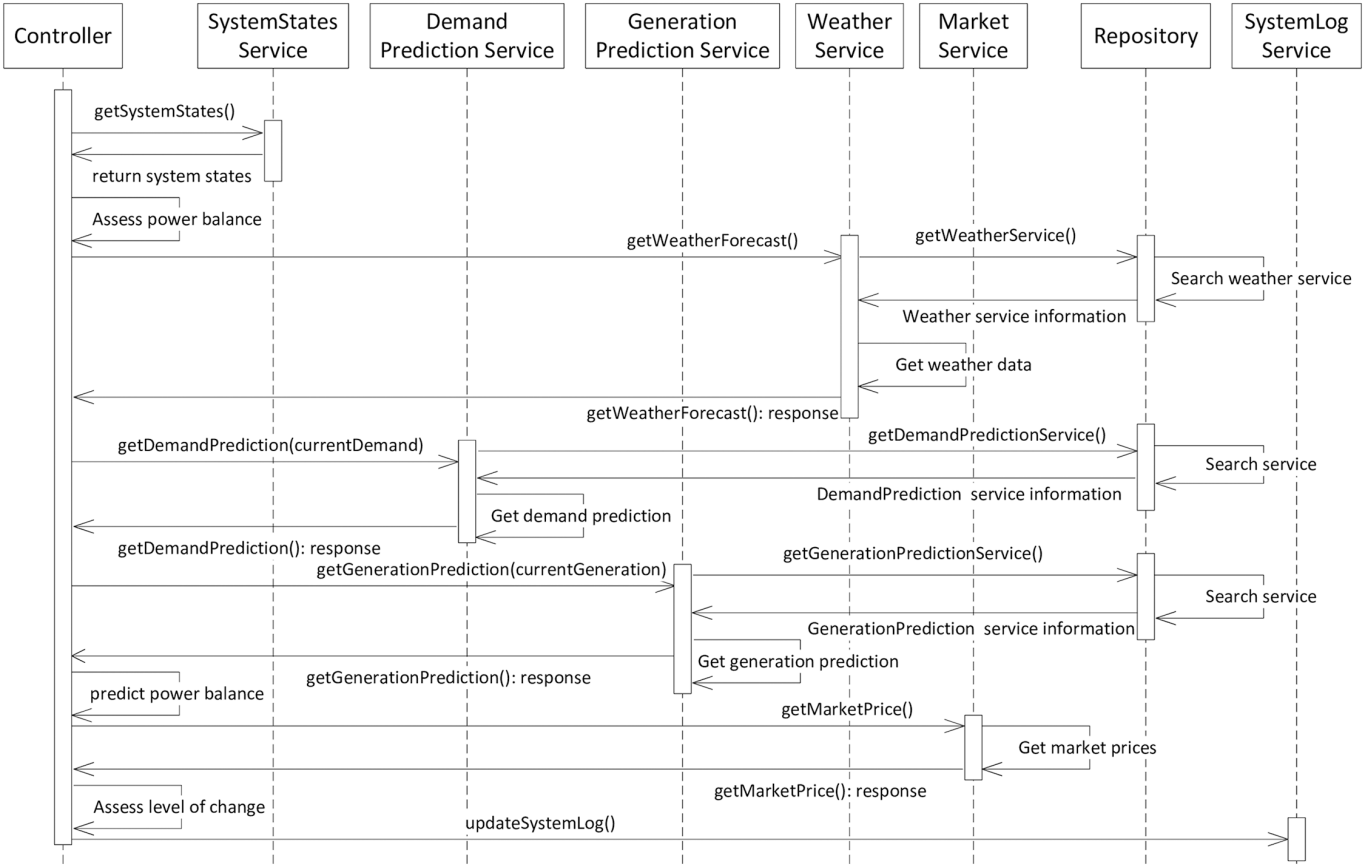
Overall system interaction view.

Fig 7 provides an overall view of service interactions. The *Controller* sends requests to *SystemStates Service* to collect information about system states from the *Demand Service*, the *Generation Service* and the *Storage Service*. A closed loop represents where the *Controller* uses the input to assess the power balance. It also sends a request to the *Weather Service* to get a weather forecast. The *DemandPrediction Service* and *GenerationPrediction Service* are then accessed to get demand and generation data. On the basis of weather forecast data, together with the demand and generation prediction data, the *Controller* is able to predict power balance. A request is also sent to the *Energy Market Service* to get the current and predicted buy and sell prices. The *Controller* checks the level of change required in case of any variation in energy balance. Finally it communicates with the *SystemLog Service* to log system states.

## Design evaluation

The validity of a case study is determined by the reliability of the results [43]. Here, ‘reliability’ has been interpreted as meaning the quality of the resulting design. We discuss the way that the evaluation of our design was performed, and then examine the conclusions that were produced from this in terms of our original research questions.

### Design of the evaluation

In discussing our research design, we noted that a key role for the evaluation process was to perform *data triangulation*, gathering different elements of evidence in order to identify how far these agreed or disagreed. The challenge was therefore to identify as many sources of data as possible. While cross-checking across the diagrams making up our design model allowed us to informally assess its completeness, we need to find a way to ‘exercise’ the model, both in terms of how it would perform (the *technology*) and how well it would meet the needs of the domain (the *application*).

To do so we chose to evaluate the case study results by employing peer review (in the form of a walkthrough) a form which could be used to evaluate both the technical content and quality of the work [44]. We considered the walkthrough technique to be appropriate for our purposes for two reasons:

- we could not find any application that could form the baseline for any comparative form of study; and
- our choice of case study topic meant that the validation process needed to employ knowledge from both the technology and application domains.

Hence, the walkthrough was performed by involving experts from the energy domain as well as software engineering.

Since the walkthrough was used for an academic purpose, which is not the usual way it is used, we considered it appropriate to employ an element of method triangulation by combining this with an action research component, allowing us to tailor the walkthrough model if necessary. Action research is ‘*cyclic*’ approach and is based on the process of *‘plan-act-reflect’* [33]. Hence, after each walkthrough session, interviews were conducted with the participants in order to collect feedback about the walkthrough process and the design presentation.

The main weakness of our choice of evaluation method is that both data and method triangulation elements draw upon the use of a shared mechanism. While this could have been reduced by using separate walkthroughs for the technology and application aspects, we concluded that the benefit of having a degree of interaction between two outweighed any disadvantages.

The details about the conduct of the walkthrough are discussed below.

**Form of review:** A walkthrough is largely qualitative in form and the documents are made available to the review committee before the evaluation. The reviewers review the document critically before the session takes place and during the review session, check-lists are used for evaluation. A set of documents that included the walkthrough protocol, the use case details, and the design model, were made available to the review team.
  We used part of the walkthrough session to present the design, and our approach was exploratory, discussion oriented and informal. This fits well with the purpose of a walkthrough as described by [45].
**Review Team and Roles:** As identified in the protocol, the roles for the walkthrough include the moderator, reviewers and the case study researcher (first author). The session was chaired by the moderator. The walkthrough protocol was sent to him prior to the session, together with the review schedule and the questionnaire. However, the use case and design documents were only sent to the reviewers.
  The expert team included two members; one from the energy engineering research group and other from computer science. To avoid bias, neither expert had previously had any involvement with any stage of the use case and design preparation. The fourth participant in the review was the case study researcher, who was responsible for presenting the design, along with the information about the use case.
**Review Schedule:** The duration for the review was two hours. This time period is considered effective for a review session [46] and so was the time period used.
**Review Procedure (First Review):** The review session was chaired by the moderator who introduced the team members and provided the detail about the session. The case study researcher provided an overview of the use case and the requirements. The main elements of the system and how they link with each other were explained. This was carried out using a whiteboard. After that, discussion with reviewers was carried out, based on the questions they raised from the documents provided.
  The moderator used the review schedule as defined in the review protocol, dividing the session into three categories; requirements, assumptions and design. At the end of each category, the questionnaire was used to ensure that all the questions listed in each category had been covered.
**Data and Record Keeping:** The review was conducted in an environment where audio and video facilities were available. A microphone was attached to each member of the team. Video cameras were allocated to each team member and also to capture the whiteboard activity. The complete session was recorded, which eliminated the need for taking notes.

The second walkthrough session was largely carried out in the same manner. However, there were some modifications including the ones that were identified through the interviews with the participants, and some that were due to unforeseen reasons.

**Review Documents:** The first walkthrough identified that there was a need for additional documents that could help with understanding issues related to both domains. For this reason, further supporting documents were prepared, which were:
  - Diagrams representing a system level view.
  - A component diagram
  - A flow chart to represent current system flow
  - A flow chart to represent part of prediction flow
  - A list of acronyms
  - A document providing a key for each notation used in the diagrams
  - An SSEZ network diagram design
**Review Team and Roles:** The team members remained the same and no changes were made in the roles. However, due to an incident on the day of the second review, the moderator was unavailable, and no arrangements could be made for another one at such short notice. However, as the review team had established its procedures during the first review, it was decided to continue to follow these without the moderator being present.
**Review Procedure (Second Review):** The session was carried out largely using the pattern employed in the previous walkthrough session

Interviews were conducted following each walkthrough session to collect feedback from the participants about the walkthrough process and about the presentation of the design. This was because:

- The walkthrough was conducted with a team that had little prior experience of reviews.
- The walkthrough was used as part of an academic exercise, which is unusual. Therefore, we considered it necessary to collect participants’ views about its effectiveness. (This provides an action research element to the review process.)
- The walkthrough involved knowledge about two different domains. It was important that the reviewers and case study researcher had the same understanding of the problem regardless of disciplines.

A semi-structured questionnaire was prepared to support the interview process. The first part was about the walkthrough process, and included the effectiveness, organisation and any improvement of the process if required. The second part was about the design presentation itself, and included the understanding of the design, its presentation and any improvements required. The interviews were also recorded.

### Outcomes from the evaluation

Here we address the two research questions that were posed at the start of the paper.

- What properties of software services need to be represented and modelled for the design of software service applications?
- How well can existing software design notations be used to describe the properties identified in answering RQ1?

The preceding sections present a relatively substantial case study of service design development. The subsequent expert evaluation, conducted over two phases, identified no major issues in terms of the viability of the design, as well as demonstrating that the set of notations used for the SOA design model provided adequate support for making and recording design decisions.

We cannot claim that our set of notations is optimum. As is common with the development of software design notations, we have borrowed from existing box and line forms in a relatively ad hoc manner as the design evolved, interpreting these as seemed most appropriate.

In terms of Table 1, these notations address most of the characteristics summarised there, apart from *delivery* and *packaging*, which are more related to implementation than design. Also, our model has taken a rather high-level view of negotiation. Arguably all of the remaining ones are part of our model in some way, and Table 9 shows how each has been represented.

**Table 9:** Representing the SOA properties.

Characteristics	Notations Used
Architecture	DFD, Component Diagram
Binding	Component Diagram, Flow Chart
Capability	Activity Diagram, Flow Chart
Composition	Class Diagram, Component Diagram, Activity Diagram, Flow Chart, Sequence Diagram
Contracts	Class Diagram, Sequence Diagram
Identity	Table, Class Diagram
Distributed Sources	Component Diagram

Table 9 therefore represents an answer to the first research question regarding the SOA properties that need to be modelled and partly answers the second question through the set of notations employed. We do not claim that this set is complete, and equally it is possible that it could also be rationalised and reduced. However, given the range of characteristics involved, it seems unlikely that realistic SOA designs could be described without using a fairly wide range of notational forms. Unfortunately there seems to be no corpus of research into how to develop a rational set of notations for describing design models for a given software architecture. Historically these seem to have evolved from experience, rather as has occurred here.

## Discussion

Here we discuss the threats to validity, consider some consequences of our experiences, and seek to draw some lessons from these.

### Threats to validity

These are connected with the way that we performed and evaluated the case study, and are discussed in terms of the usual categories.

**Construct Validity** For the case study itself, the main threat is our choice of a single-case form, necessitated by the size of the task involved. Further case studies are needed to confirm that this was a ‘typical’ case.
  In terms of our evaluation, walkthroughs have been used since the 1970’s and 1980’s [47]. Overall, there appears to be a lack of empirical studies on the experience of conducting walkthroughs and little guidance about their use for the purpose of design evaluation.
  We therefore made use of the guidelines available at [48] and mentioned in [46], tailoring these to write the protocol for the walkthrough. We also employed elements of an action research approach by gathering feedback from the participants about the walkthrough process as suggested in [49]. This provided some confidence that the form of the protocol and the walkthrough process were appropriate, as did the participant feedback obtained through the questionnaire.
**Internal Validity** For the case study this was addressed by using both method and data triangulation.
  - *Method triangulation* involved constructing an operational model in the form of a use case; producing a design by using existing design techniques and knowledge; and performing a walkthrough to check the validity of the use case and the SOA design model.
  - *Multiple data sources* included formal and informal interviews with application domain engineers, analysis of technical papers, and other supporting documents.
  The evaluation involved human participation, and the participants had backgrounds in different domains and different levels of knowledge about the problem under discussion. The following possible sources of bias were identified as below.
  - *Selection of Experts*: Two experts were involved in the study, one from each domain. This could be considered to create a risk of overlooking any major issue during the review. However, the questionnaire, which was verified by the second author (DB), was employed to ensure we did not ignore any important point.
    The SSEZ use case was also constructed with the help of energy engineers and feedback was taken during requirements gathering to verify that what is written was understood by the author and meaningful to engineers. This does not violate the walkthrough guidelines, where it is suggested that the number of experts involved can be restricted to two. The selection of an expert was done by the members of the energy research group who knew about our work. The expert from computer science was selected on the basis of their experience with SOA as well as with software design in general.
  - *Involvement of Experts*: Neither of the experts who took part in the review was involved at any stage during the preparation of the use case and design.
  - *Data Consistency*: There is a threat to data consistency when observers are taking notes, which can be controlled by assigning more than one observer for the inspection [49]. We addressed this issue by keeping audio and video records of all sessions, which also helped with analysing the sessions and viewing the discussion in its context.
  - *Analysis*: The analysis of the walkthrough was done by the first author alone, but checked with the second author.
**External Validity** For the case study as a whole, this is largely a consequence of its design. Our case study is a single-case design, a choice that raises the issue of bias and generalisation when considering any outcomes. Bratthall & Jørgensen [50] have noted that use of multiple data sources in an exploratory case study can make it more trustworthy than one that is based on single source of data. We have used multiple sources of data where possible, but despite this, it would still be unwise to generalise too far from this one case.

### What the outcomes tell us about designing with software services

The value of an exploratory case study such as this comes largely from exploring the bounds of the problem—in our case, that of designing a system that would be created largely from software services. While the evaluation of our design was based upon expert judgement rather than implementation, it can still be argued that we have been able to explore the issue of what needs to be addressed in an SOA design (Research Question 1) and demonstrated how this can be modelled (Research Question 2).

Our discussion so far has addressed methodological choices, such as the use of case study research, and of performing design evaluation through the use of walkthroughs. Here, we return to the issue of designing SOA systems., and in particular, how such a design might be modelled.

Our eventual set of notations, summarised in Table 9, represents a set of choices based upon a compromise between the need to model particular properties, and also to have the convenience of using familiar modelling tools and notations. While we did employ notations from the UML, these were not interpreted in terms of objects. Indeed, both Sequence Diagrams and Activity Diagrams are concerned with modelling interactions, regardless of the forms of software element involved in the interactions, and therefore can be considered as architecturally neutral.

However, what we can conclude is that while it is not essential to devise new notations for modelling the properties of service-based systems, there might well be some benefits from rationalising and improving upon the ones that were used. Both the Class Diagram and the Component Diagram forms tend to reflect the object model in terms of their form—suggesting that modellers might find it helpful to have more distinctively service-oriented forms available. This might be particularly relevant when making decisions about such characteristics as system architecture (where neither the DFD nor the Component Diagram reflect the hierarchical aspects well), and contracts between services, which do involve some reinterpretation of the Class Diagram. Indeed, in terms of future research, it might be possible to reuse this case study in order to explore how well other forms of notation might work, and to compare them with those from the UML.

Recent research suggests that the UML is rarely used for developing design models, although it might sometimes be used for recording them [51]. One possible reason leading to this situation could be the complexity and size of the UML. In this study we have demonstrated that an SOA design solution can be modelled using a much smaller set of notations (we use seven in Table 9), and also we only need to use a limited set of notational features from these.

For the purposes of the case study therefore, this set of notations would appear to provide adequate support for developing and exercising the form of design model needed to describe an SOA system. However, given the risk of confusion arising from reusing notations developed around a significantly different architectural style (objects), there would be benefits in devising new forms of notation.

### Some lessons from the case study

While the object paradigm has proved to be a very powerful vehicle for system implementation and reuse, its inherent complexity does involve complex decision-making and trade-offs in design.

Our two research questions were essentially formulated to determine whether such a situation also held for SOA design. So here we summarise some key insights that we obtained from our case study, subject of course to the caveats identified at the start of this section.

- *Lesson 1: Modelling of SOA designs requires less complex forms than those used for the object-oriented architecture.* This is evident from Table 9, which essentially summarises the answers to both research questions. We have not really addressed the issue of how to integrate the different viewpoint elements in the model, but we were able to produce a design model using fewer notations than would be considered as normal for object-oriented designs.
- *Lesson 2: There appears to be no recognised procedures for creating a set of ‘design notations’ appropriate for an architectural style.* Despite extensive search and consultation, we were unable to identify any practices that could be used to aid with developing a set of notations from the characteristic properties of a particular architectural style. Historically, design notations would seem to have evolved in an ‘ad hoc’ manner, with little or no consideration of cognitive aspects.
- *Lesson 3: The SOA paradigm would benefit from a unified set of modelling notations.* Our study of the literature in seeking to identify SOA characteristics, together with the experiences from the case study, suggests that there is a need for the community to identify and promote an effective set of modelling notations, both for sharing of ideas, and also to assist when developing larger-scale designs. Indeed, while the UML is open to technical criticism, it has nonetheless been useful in providing a common vehicle for for describing and exchanging ideas about design.

## Conclusion

This study was conducted by employing a multi-method approach: first conducting a mapping study to find out what characteristics are associated with SOA; translating these to the attributes of the case study taken from energy engineering; constructing a design by employing existing notations; and finally evaluating them through expert review. Hence it provides a chain of evidence to address the issues arising in service-based application design.

Case studies used in designing service based systems largely make use of toy examples, whereas here we have presented a moderately large-scale exploratory case study of SOA design that is based upon a real-world problem. While the problem is drawn from the engineering domain, it does possess some ‘business’-related characteristics too, and offers useful insight into SOA design challenges.

We have been able to make a good assessment of the key characteristics that need to be addressed in the SOA design process. The design solution developed in the case study has also allowed us to identify and model a set of key characteristics and interactions. In doing so, we have confined ourselves to using existing modelling notations, although with some reinterpretation where relevant.

We also identified that, while designing service based applications, in addition to knowledge of the application and software domains, the current and future business policies should be considered. The choice between being a service provider or a service consumer plays an important role in designing service applications.

### Study limitations

The case study research method has the implicit limitation that the results cannot be generalised, at least, for single case studies such as this. Our choice of a case study taken from energy engineering also means that real time constraints need to be considered, which might not be required in more business centred systems.

### Future directions

In terms of future research, we would suggest that the modelling and representation of such systems is therefore a key area meriting a fuller investigation. We would also suggest that our case study could itself be employed to provide a benchmark problem that is relatively accessible to a non-domain expert and also large enough to present a realistic challenge, and that our design model could also provide a baseline for comparing the use of other modelling forms.
